# King Oyster Mushroom, *Pleurotus eryngii,* Inhibits Microglia Activation via the Interplay of NLRP3 to Alleviate Neuroinflammation

**DOI:** 10.3390/nu18101495

**Published:** 2026-05-08

**Authors:** Isabelle Aurore Hininger-Favier, Derek R. Fisher, Ahcene Boumendjel, Barbara Shukitt-Hale

**Affiliations:** 1 Laboratory of Fundamental and Applied Bioenergetics (LBFA), Institut National de la Sante et de la Recherche Médicale (INSERM) Unit 1055, Université Grenoble Alpes, 38000 Grenoble, France; 2 United States Department of Agriculture, Agricultural Research Service, Human Nutrition Research Center on Aging, Tufts University, Boston, MA 02111, USA; derek.fisher@usda.gov (D.R.F.); barbara.shukitthale@usda.gov (B.S.-H.); 3 Radiopharmaceutiques Biocliniques (LRB), Institut National de la Sante et de la Recherche Médicale (INSERM) Unit 1039, Université Grenoble Alpes, 38000 Grenoble, France; ahcene.boumendjel@univ-grenoble-alpes.fr

**Keywords:** mushroom, microglia, neuro-inflammation, NLRP3

## Abstract

**Background:** Mushrooms have gained attention for their potential to improve brain health. We evaluated extracts of king oyster mushroom, as well as two of its bioactive compounds—ergothioneine (ERG) and *N*-acetyltryptamine (NAT)—for their ability to prevent microglia activation by reducing neuroinflammation and oxidative stress. **Methods**: HAPI microglial cells were pretreated with king oyster extracts (crude powder, acetone, ethanol, and methanol extracts at 100 μg/mL) and pure bioactive molecules of ergothioneine (ERG, 500 μM) and *N*-acetyl-tryptamine (NAT,50 μM) before stimulation with LPS. The effects on nitrite; TNF-α; and expressions of the inflammatory proteins iNOS, NOX2, and NLRP3 were compared with those of a blueberry extract (BB, 500 μg/mL) as a positive control. **Results:** All extracts and bioactive molecules significantly reduced nitrite production, similar to the BB. Overall, the best results for reducing inflammation and inflammatory protein expression were obtained with the extracts rich in NAT (acetone and ethanol), as well as pure NAT. Furthermore, through their inhibitory target effect on NLRP3, these two extracts and the bioactive compounds (NAT and ERG), like BB, are attractive therapeutic molecules to reduce mood disorders related to brain aging, due to evidence of enhanced Nucleotide-binding oligomerization domain-like receptor family pyrin domain-containing-3 (NLRP3) inflammasome activity in common neurodegenerative diseases. Further interventional studies are needed to confirm mushrooms’ brain health properties.

## 1. Introduction

With the increase in life expectancy, healthy aging has become a major concern and a global public health priority [1]. In the absence of effective pharmacological drugs, physical activity and healthy nutrition are the gold standards for aging in good cognitive health. It has been demonstrated that a higher adherence to a healthy diet, such as the Mediterranean diet, can lower the risk of dementia, underlining the importance of the quality of diet in preventing brain disorders [2]. Recently, edible mushrooms have gained attention as a healthy food because they are a valuable source of bioactive molecules that can enhance immune function and reduce susceptibility to disease [3,4]. A recent review [5] concluded that consuming ~120–150 g of mushrooms per week appeared sufficient to significantly reduce the risk of cognitive impairment, depression, and all-cause death. However, according to the same report, it is important to elucidate the molecular mechanisms involved in the improvement of cognition by mushroom intake.

*Pleurotus eryngii*, also known as the king oyster mushroom, is an edible and medicinal fungus that is rich in nutrients and bioactive compounds. Given its significant presence in European and American markets compared to other Asian medicinal mushrooms—such as shiitake, reishi, or lion’s mane—we prioritized its study. Additionally, we have previously demonstrated that NAT, a serotonin-like molecule found in this mushroom, functions as an acetylcholinesterase inhibitor [6], underscoring its therapeutic potential for cognitive health [7]. In addition to its notable dietary fiber and vitamin contents and its strong capacity to absorb and incorporate minerals into organic compounds, most prior research on *P. eryngii* has focused only on its polysaccharide-rich extracts [8]. Moreover, another bioactive molecule present in mushrooms is ergothioneine (ERG), and the king oyster mushroom is one of the richest in ERG among mushroom species [9]. ERG has recently gained attention for its potential beneficial effects on aging [10], including neurological disorders [11]. ERG is a sulfur-containing derivative of the amino acid histidine, has a high redox potential due to its stable thione structure [9,12], and confers cytoprotectant properties [13]. A specific transporter for ERG (named carrier protein organic cation transporter novel type-1 (OCTN-1), or SLC22AA4) is present in the brain [12,14], including the microglia [15], enabling ERG to cross the blood–brain barrier. Interestingly, it has been reported that ERG levels in the blood decline beyond 60 years of age and decline faster in those with cognitive impairment, suggesting that low blood ERG levels may be associated with neurological diseases [16]. These observations led us to consider that ERG might be an important bioactive molecule that could promote healthy brain aging by modulating neuroinflammation.

It is now widely acknowledged that neuroinflammation is mediated by microglia, which release reactive oxygen species (ROS) when activated, as well as increasing the expression of inflammatory cytokines such as NOX2 (producing O_2_^−^), as a hallmark in the pathogenesis of the aging brain, neurodegenerative diseases, and depression [17,18,19,20]. Microglia activation promotes nitric oxide synthase (iNOS) induction and subsequent NO and nitrite production [21]. Lipopolysaccharide (LPS), a crucial bacterial component [22] that is largely used in experimental studies as a model to activate microglia, has also more recently been described as an activator of the nucleotide-binding oligomerization domain-like receptor family pyrin domain-containing-3 (NLRP3) inflammasome [23]. Increasingly more studies indicate that the regulation of the inflammasome signaling pathway is crucial for the homeostasis of inflammatory responses, especially in the CNS, where NLRP3 is described as a major contributor to the neuroinflammatory processes leading to brain disorders such as memory impairment and depression [24,25]. Therefore, the inhibition of NLRP3 inflammasome activation has been proposed as a potential therapeutic strategy for neuroinflammation-related diseases [25], and future treatment strategies should target such diseases through microglia activation [24,25,26].

To the best of our knowledge, no studies have evaluated the effects of *Pleurotus eryngii* and its bioactive compounds, NAT and ERG, on activated microglia and the NLRP3 inflammasome. Therefore, in the present study, we examined the hypothesis that king oyster mushroom might reduce the release of pro-inflammatory factors by activated microglia, and that part of this effect might be mediated by its bioactive compounds NAT and ERG. Thus, we compared the anti-inflammatory effects of different *P. eryngii* extracts (acetone, ethanol, and methanol) with those of the whole mushroom and its isolated bioactive compounds (NAT and ERG). All conditions were compared with a blueberry extract used as a positive control. Indeed, previous experiments, including human studies from our group and others [27,28,29,30], have shown that BBs are antioxidants with anti-inflammatory properties against activated microglia, and that dietary intake of BB improves age-related declines in brain function.

## 2. Methods

### 2.1. Chemicals and Reagents

The commercial compounds used in this study were purchased from Sigma-Aldrich (St. Louis, MO, USA), unless otherwise noted. The solvents used for the preparation of the extracts were HPLC-grade and were purchased from Carl Roth (Lauterbourg, France).

### 2.2. Preparation of King Oyster Extracts

The mushroom material (collected in 2020 in Algeria, Oued El Atmania region) was washed with water, dried at room temperature, and then ground to a fine powder, referred to here as K-crude. In total, three extracts were prepared. The extraction conditions differed in terms of mushroom mass, extraction time, and solvent volume. These parameters were chosen to ensure high extraction yield without degradation of the extract components.

Acetone extract (K-AC): Mushrooms (7.2 g) were mixed with 300 mL of acetone. The mixture was stirred at 50 °C for 16 h. The solution was filtered over filter paper and then evaporated under reduced pressure at 50 °C to yield a beige–yellow solid (350 mg). This extract was the richest in NAT, estimated at around 0.015% following its isolation by preparative TLC and identification by ^1^H NMR experiments.

Ethanol extract (K-ET): Ten grams of mushroom powder was mixed with 300 mL of ethanol. The mixture was stirred at 50 °C for 36 h. The solution was filtered over filter paper and then evaporated under reduced pressure at 50 °C to yield a beige–yellow solid (945 mg). NAT was detected at a lower concentration compared to the K-AC extract (around 0.001%).

Methanol extract (K-ME): Mushroom powder (20.5 g) was mixed with 400 mL of methanol. The mixture was stirred at room temperature for 2 h and then heated at 50 °C for 26 h. The solution was left to return to room temperature, and then it was filtered over filter paper before being evaporated under reduced pressure at 50 °C to yield a brown semi-solid product (2.56 g). MeOH was used as a polar solvent due to its ability to extract polar compounds such as polyphenols and polysaccharides. The three extracts (K-AC, K-ET, and K-ME) were resuspended in ETOH and stored at −20 °C until use. The mushroom powder (K-crude) was only soaked in cell media and agitated overnight at room temperature. The mixture was then centrifuged for 10 min at 10,000 rpm and filtered before being stored at −20 °C.

The pure NAT (*N*-acetyltryptamine; Cayman Chemicals, Ann Arbor, MI, USA) and ERG (L-(+)-ergothioneine; Target Mol, Boston, MA, USA) were resuspended in distilled water and stored at −20 °C until use. Stock solutions of NAT (MW = 202.3 g/mol) and ERG (MW = 229.3 g/mol) were prepared in distilled water at concentrations of 1 and 10 mM, respectively, and stored at −20 °C. Working solutions were freshly prepared by diluting the stock solutions to obtain final concentrations ranging from 0 to 100 μM for NAT and from 0 to 1 mM for ERG. These dilutions were made in cell culture medium in 50 mL Falcon tubes, which were then used to resuspend the cells. Subsequently, 1 mL of cell suspension was distributed in duplicate into 12-well plates.

### 2.3. Cell Culture and Treatments

Highly aggressively proliferating immortalized (HAPI) microglial cells represent a reliable and practical model for investigating neuroinflammatory processes in vitro [31,32]. HAPI rat microglial cells (generously provided by Dr. Grace Sun, University of Missouri, Columbia, MO, USA) were maintained in Dulbecco’s modified Eagle’s medium (DMEM, Thermo Fisher Scientific, Carlsbad, CA, USA) supplemented with 10% fetal bovine serum, 100 U/mL penicillin, and 100 μg/mL streptomycin at 37 °C in a humidified incubator under 5% CO_2_. For the cell cultures and treatments, we followed the same methods previously described in [27]. For the experiments, cells were split into 100 mm plates at a seeding density of 2.5 × 10^6^ cells/well and pretreated with DEM containing the K extracts and bioactive compounds (ERG, NAT) or BB extract as a positive control. Freeze-dried BB extract was prepared as described previously [33] and diluted in medium to a concentration of 0.5 mg/mL. The chosen dose of the positive control was 0.5 mg/mL blueberries (BB), which significantly attenuates LPS-induced inflammation in microglia and is not cytotoxic [27,28]. For pretreatments, cells were incubated for 24 h in the absence (control) or presence of the mushroom extracts diluted in media at different concentrations (K, K-AC; K-ET; K-ME: 0–0.4 mg/mL), or of the bioactive ingredients ERG (0–500 μM) and NAT (0–100 μM), to test viability. Then, we selected the minimal dose with a beneficial effect on nitrites (K extracts: 100 μg/mL; ERG: 500 μM; NAT: 50 μM). On day 2, the pretreatment media were removed, and cells were split into 12-well plates and stimulated or not (control) with a bacterial endotoxin and inflammatory stressor, lipopolysaccharide (LPS, Sigma-Aldrich, St. Louis, MO, USA), in serum-free DMEM without phenol red at 0 or 200 ng/mL overnight (16–18 h). This dose has been shown to induce stress signaling without reducing cell viability [30]. For each of the conditions, treatments were performed twice, and the number of independent experiments was between 3 and 5. Furthermore, we also observed an effect of LPS on HAPI microglial cell activation, as assessed by a significant increase in Iba1 expression, a hallmark of microglia activation (C = 4.55 ± 2.25 vs. LPS = 14.32 ± 6.81 relative band density (×10^5^); *n* = 3; *p* < 0.01). This analysis was performed as described below for Western blotting using a primary antibody for rabbit anti-Iba-1 at 1:500 (Wako chemicals, #01919741).

### 2.4. Cell Viability

Cell viability was assessed using the Live/Dead Cellular Viability/Cytotoxicity Kit (Thermo Fisher). In brief, microglial cells were seeded onto 12-well plates, and the experimental treatments were performed as described above. After pretreatment at the ranges discussed above for the K extracts (0–400 mg/mL), ERG (0–1 mM), and NAT (0–100 μM), microglial cells were dually stained with two probes (Calcein AM for live cells, colored in green; ethidium homodimer-1 for dead cells, colored in red) to enable the simultaneous determination of live and dead cells in a sample, according to the manufacturer’s instructions. Fluorescent images of the cells were captured with a Nikon Eclipse Ti2 inverted fluorescent microscope. The number of independent experiments was *n* = 3; the numbers of live and dead cells were manually counted for each image using Nikon Elements software (version 5.42.04), and data were expressed as the percentage of viable cells. Viability was assessed for K extracts (50, 100, 200, 400 μg/mL), crude K powder (10, 50, 100, 200, 400 μg/mL), ERG (0.125, 0.25, 0.5, 1 mM), and NAT (1, 5, 25, 50, 100 μM).

### 2.5. Nitrite Quantification

Following LPS stimulation of the HAPI cells, the supernatant from each well was removed and stored at −20 °C until use. Nitrite (NO_2_^−^) concentrations were determined using the Griess colorimetric method based on a diazotization–coupling reaction. A stock solution of sodium nitrite (NaNO_2_) (1 mM) was prepared in deionized water and further diluted to obtain working standards (0–100 µM). A volume of 100 µL of standards or cell supernatants was transferred to a 96-well plate. Then, 25 µL of the Griess reagent was added and incubated in the dark for 15 min. The absorbance was measured at 540 nm using a microplate reader (or spectrophotometer). A reagent blank (deionized water plus Griess reagents) was used for baseline correction. Nitrite concentrations were calculated from a standard calibration curve generated using sodium nitrite standards (y = 0.0056x + 0.062). The results were expressed as µM or mg/L NO_2_^−^. All measurements were performed in duplicates of *n* = 3 to 5 independent experiments.

### 2.6. TNF-α Enzyme-Linked Immunosorbent Assay

Secretion of the inflammatory cytokine TNF-α was quantified using an ELISA according to the manufacturer’s instructions (Invitrogen, Carlsbad, CA, USA). Therefore, 50 μL of cell-conditioned supernatant or provided protein standards was added in duplicate to a 96-well plate and incubated for 2 h. Following washing, a peroxidase-conjugated detection antibody was added to each well and incubated for 1 h. The wells were washed, and substrate solution was added to each well for 15 min, followed by stop solution. Absorbance was read at 450 nm, and the TNF-α concentration in each sample was calculated from the linear equation derived from the standard curve of known concentrations of the cytokine. Values were normalized by total protein levels in cell lysates quantified with the DC protein assay (BioRad, Hercules, CA, USA).

### 2.7. Western Blots

Based on the viability and nitrite results, we selected concentrations of 100 μg/mL of mushroom and K extracts, 50 μM NAT, and 500 μM ERG. As previously described [27], cells were washed in ice-cold PBS, resuspended and lysed by agitation in CelLytic-M Cell Lysis Reagent (Sigma-Aldrich) containing phenylmethylsulfonylfluoride (PMSF, 10 ug/mL), and centrifuged at 18,000× *g* for 10 min at 4 °C to yield the resultant supernatant lysate, which was used for blotting after the total protein was quantified with the DC protein assay (BioRad, Hercules, CA, USA). Western blots were performed as described previously [27]. Primary antibodies for iNOS (MilliporeSigma, Burlington, MA, USA), NOX2 (Santa Cruz, Dallas, TX, USA), and NLRP3 (Novus Biologicals, Centennial, CO, USA) were used at 1:1000 dilutions for incubation overnight at 4 °C. Glyceraldehyde 3-phosphate dehydrogenase (GAPDH, Santa Cruz, Dallas, TX, USA) was used as a protein loading control marker. The signal was detected using an electrochemiluminescence (ECL) detection kit (Bio-Rad, Hercules, CA, USA). The optical density of antibody-specific bands was analyzed using the VisionWorks LS image acquisition and analysis software (UVP, version 8.1.2, Upland, CA, USA). Data are expressed as the relative mean band density ± SEM.

### 2.8. Statistical Analysis

Data were presented as the mean ± SEM. Between three and five independent experiments were conducted. Microplate assays (proteins, nitrite, TNF-α) were performed in duplicate, and the results were averaged. Statistical analyses were performed using analysis of variance (ANOVA) with GraphPad Prism 8.0 software (San Diego, CA, USA). One-way ANOVA was used for the viability data, while two-way ANOVA was used in experiments for comparing the control and LPS conditions. Post hoc tests, when either the main effect or interaction was significant, were performed using Fisher’s least significant difference (LSD) test. Results were considered to be statistically significant if the observed significance levels with treatment were * *p* < 0.05, ** *p* < 0.01, *** *p* < 0.001, or **** *p* < 0.0001.

## 3. Results

### 3.1. Viability

**Figure 1:**
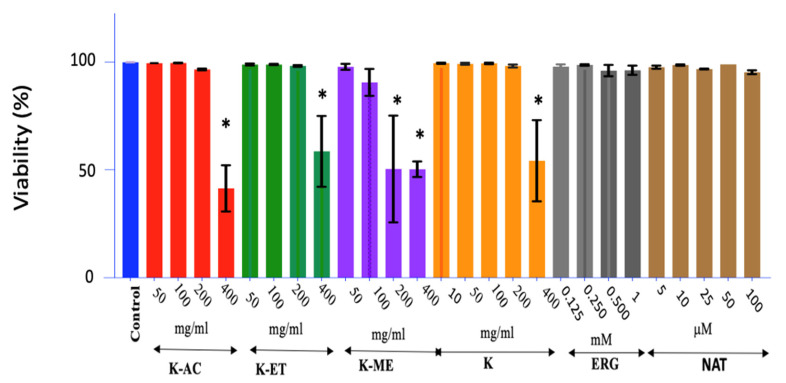
HAPI microglial cell viability after treatment with mushroom extracts or the bioactive molecules ERG and NAT. Cell viability was measured via Live/Dead assay. Effects of different concentrations of mushroom extract (K-AC; K-ET; K-ME), mushroom powder (K), ERG, or NAT on HAPI cells were assessed after 24 h of treatment. Data are expressed as the mean ± SEM of independent experiments (*n* = 3); * *p* < 0.05 vs. control.

The cell viability was 97% on average for the K-AC and K-ET extracts and K-crude at 200 μg/mL, but it dropped below 60% at 400 μg/mL (Figure 1) (*p* < 0.001). The K-ME extract was more cytotoxic, with a significant decrease in viability at 200 μg/mL (*p* < 0.05). NAT and ERG were not cytotoxic at the doses assessed here.

### 3.2. Extracellular Nitrite (NO_2_^−^) Release

**Figure 2:**
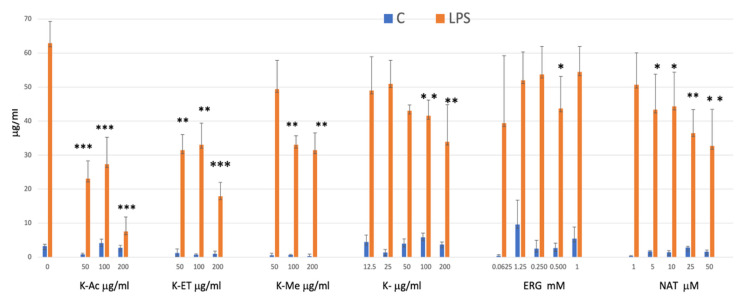
Nitrite production after HAPI microglia were pretreated (24 h) with king oyster extracts Nitrite production after HAPI microglia were pretreated (24 h) with king oyster extracts, with K-AC (50–200 μg/mL), K-ET (50–200 μg/mL), K-ME (50–200 μg/mL), K (50–200 μg/mL), ERG (0.0625–1 mM) and NAT (1–50 μM) followed by a treatment with LPS for 16 h. Data are presented as the mean ± SEM; *n* = 5 independent experiments; * *p* < 0.05, ** *p* < 0.01, *** *p* < 0.001compared with LPS control. All of the pretreatments without LPS were no different from the control.

**Figure 3:**
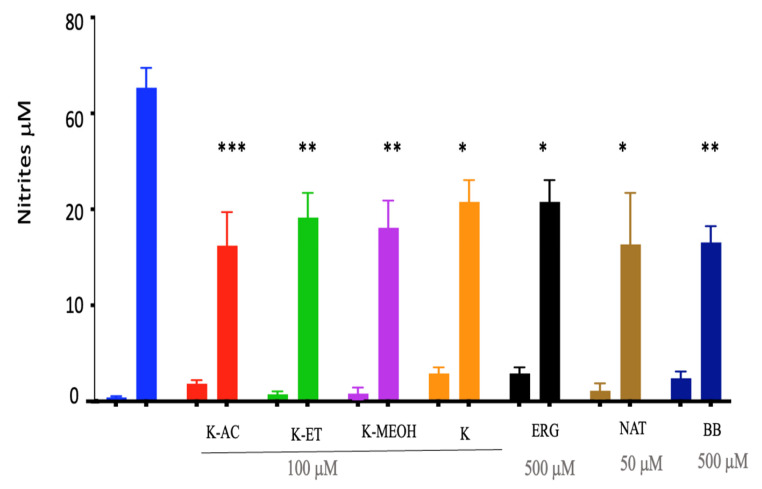
Nitrite production after HAPI microglia were pretreated (24 h) with selected concentrations of mushroom extracts (100 mg/mL), the bioactive molecules ERG (500 μM) or NAT (50 μM), or blueberries (500 mg/mL), followed by overnight treatment with LPS. Data are presented as the mean ± SEM; *n* = 5 independent experiments; * *p* < 0.05, ** *p* < 0.01, *** *p* < 0.001. All of the pretreatments without LPS were no different from the control.

The release of nitrite by microglia under LPS stimulation is well known and was confirmed here; however, the effects of king oyster mushroom, its extracts, and its bioactive molecules ERG and NAT on nitrite production were unknown. Therefore, we assessed nitrite production for the range of doses that did not affect viability. Pretreatments with extracts or bioactive molecules had no effect on nitrite production compared to the control, but they prevented nitrite release in a dose-dependent manner after LPS-induced microglia activation, for all compounds except ERG (Figure 2). From these results, we selected the lowest common dose associated with a significant effect on nitrite production—i.e., 100 μg/mL for extracts and the crude mushroom, 500 μM for ERG, and 50 μM for NAT (Figure 3)—for the subsequent experiments. Then, all of these selected concentrations were compared with one another and with BB at 500 μg/mL using ANOVA. There were significant effects of LPS (F_1,56_ = 373.1, *p* < 0.0001), treatment (F_7,56_ = 3.79, *p* < 0.01), and LPS × treatment interaction (F_7,56_ = 5.31; *p* < 0.0001). After LPS stimulation, all pretreatments significantly reduced nitrite production induced by LPS in a similar manner as the positive control, although there were discrepancies in the statistical power (K-AC had the highest significance (*p* < 0.001), while the pure compounds NAT and ERG had the lowest (*p* < 0.05)).

### 3.3. TNF-α Analysis

**Figure 4:**
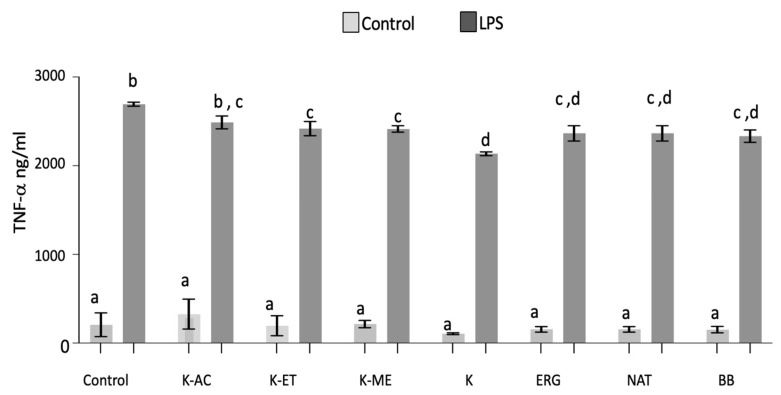
Production of extracellular TNF-α (pg/mL) in HAPI microglial cells pretreated with K-AC (100 μg/mL), K-ET (100 μg/mL), K-ME (100 μg/mL), K (100 μg/mL), ERG (500 μM), NAT (50 μM), and BB (500 μg/mL) as a positive control, before and after overnight treatment with LPS. The data were assayed by ELISA, and the values are expressed as the mean ± SEM. Treatment groups were compared with a level of significance at *p* < 0.05; *n* = 3 independent experiments. Data not sharing the same letter are significantly different.

There were significant main effects of LPS (F_1,30_ = 282.9, *p* < 0.0001) and treatment (F_7,30_ = 2.76, *p* < 0.05) on TNF-α production in microglia. Post hoc analyses showed that LPS significantly increased TNF-α levels compared with non-stressed conditions (*p* < 0.0001, Figure 4). All pretreatments significantly attenuated LPS-induced TNF-α expression (*p* < 0.05), except for the K-AC extract, which exhibited a non-significant decrease compared with LPS treatment. Interestingly, despite this lack of statistical significance, TNF-α levels in the K-AC group were no longer significantly different from its non-stressed control, and all pretreatments were similar to the LPS positive control (BB).

### 3.4. Western Blot Analysis

**Figure 5:**
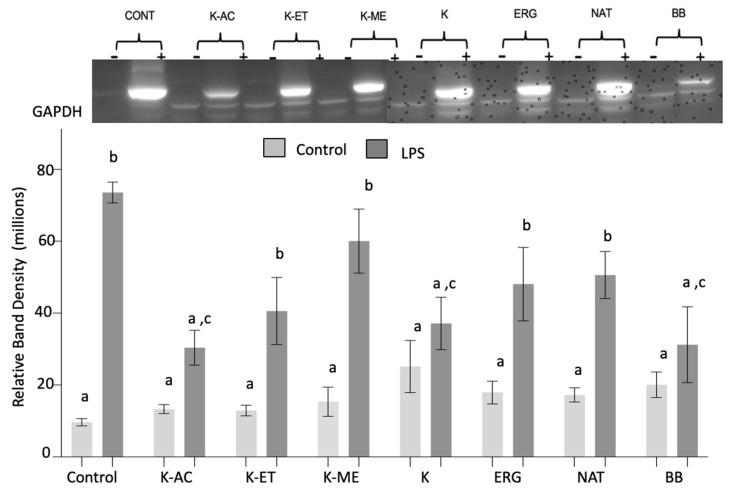
Expression of iNOS in HAPI microglial cells pretreated with K, with K-AC (100 μg/mL), K-ET (100 μg/mL), K-ME (100 μg/mL), K (100 μg/mL), ERG (500 μM), NAT (50 μM), and BB (500 μg/mL) as a positive control, before and following LPS treatment. Data are expressed as the mean ± SEM of the immunoreactive band density per 20 μg of total protein, as measured by Western blot (Top). *n* = 4 independent experiments. Data not sharing the same letter are significantly different at *p* < 0.05.

*iNOS expression*: There were significant main effects of LPS (F_1,46_ = 79.75, *p* < 0.0001), a trend for treatment (F_7,46_ = 2.01, *p* = 0.07), and an LPS × treatment interaction (F_7,46_ = 3.20, *p* < 0.01). Post hoc testing showed that LPS significantly increased iNOS expression compared to non-stressed conditions (*p* < 0.0001), while the pretreatments did not affect iNOS expression in non-stressed cells (Figure 5). The results showed a significant decrease in LPS-induced iNOS expression when cells were pretreated with K-AC, K-crude, or BB (*p* < 0.01), with the strongest statistical effect for K-AC (*p* < 0.001, *p* < 0.01, and *p* < 0.01, respectively). Additionally, K-AC, K-crude, and BB showed no significant differences in iNOS expression following LPS compared with no LPS after these treatments (Figure 5). Conversely, pretreatment with K-ET, K-ME, or the pure molecules NAT and ERG did not reduce LPS-induced iNOS expression.

**Figure 6:**
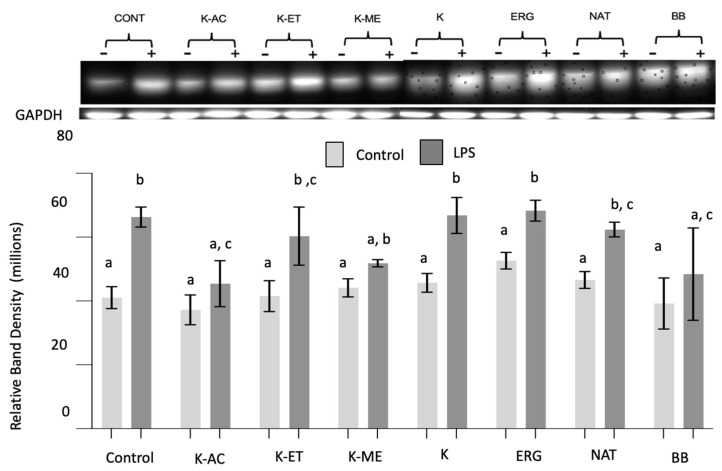
NOX2 expression in HAPI microglial cells treated with K-AC (100 μg/mL), K-ET (100 μg/mL), K-ME (100 μg/mL), K (100 μg/mL), ERG (500 μM), NAT (50 μM), and BB (500 μg/mL) as a positive control, before and following LPS treatment. Data are expressed as the mean ± SEM of the immunoreactive band density per 20 μg of total protein, as measured by Western blot (Top). *n* = 4. Data not sharing the same letter are significantly different at *p* < 0.05.

*NOX2*: There were significant main effects of LPS (F_1,40_ = 29.61, *p* < 0.0001) and treatment (F_7,40_ = 2.30, *p* < 0.05) on NOX2 expression. Post hoc testing showed that LPS significantly increased NOX2 expression compared to non-stressed conditions (*p* < 0.001) (Figure 6). Exposure to K-AC or BB, which significantly reduced LPS-induced NOX2 overexpression, as did K-ME and NAT, were no longer significantly different from their respective pretreated controls, indicating a restoration toward baseline levels. In contrast, K-ET, K, and ERG did not significantly reduce LPS-induced NOX2 expression, as their levels remained significantly higher than their pretreated controls and were not different from LPS-treated cells without pretreatment.

**Figure 7:**
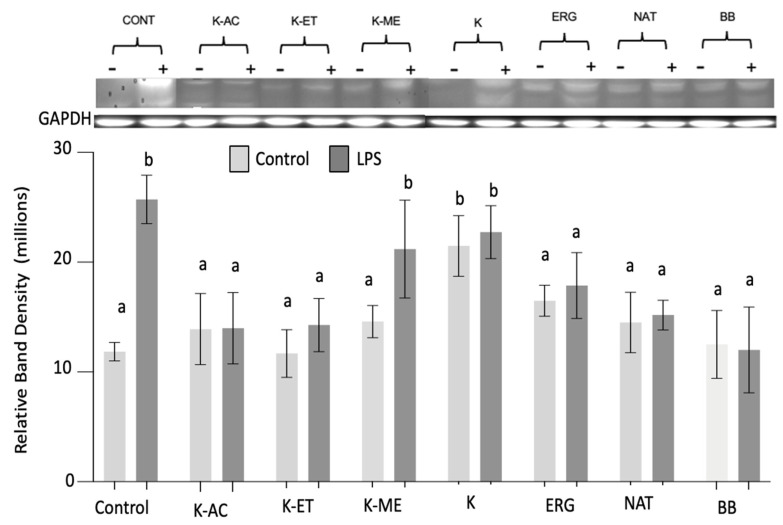
NLRP3 expression in HAPI microglial cells treated with K-AC (100 μg/mL), K-ET (100 μg/mL), K-ME (100 μg/mL), K (100 μg/mL), ERG (500 μM), NAT (50 μM), and BB (500 μg/mL) as a positive control, before and following LPS treatment. Data are expressed as the mean ± SEM of the immunoreactive band density per 20 μg of total protein, as measured by Western blot (Top). *n* = 5 separate experiments. Data not sharing the same letter are significantly different at *p* < 0.05.

*NLRP3*: NLRP3 showed significant main effects of LPS (F_1,67_ = 5,82, *p* = 0.02) and treatment (F_7,67_ = 2.55, *p* < 0.05). Post hoc testing showed that LPS significantly increased NLRP3 expression (*p* < 0.001) in control microglia. All of the pretreatments had no effects on NLRP3 expression in the absence of LPS, except for a significant increase with the crude K (*p* < 0.01). After LPS treatment, the NLRP3 expression significantly decreased following pretreatment with K-AC and K-ET (*p* < 0.01), as well as ERG and NAT (*p* < 0.05), but not with K-ME or K-crude. Furthermore, the decreases induced by pretreatments were stronger, as they were no longer different than their pretreated control cells. These effects were similar to those of the positive control BB (*p* < 0.01) (Figure 7).

## 4. Discussion

We aimed to investigate the effects of king oyster mushrooms, their extracts, and their pure compounds (ERG and NAT) in preventing neuroinflammation induced by microglia. As expected, we observed that hallmarks of neuroinflammation such as nitrite production, TNF-α, and INOS, NOX2, and NLRP3 expression were increased following LPS activation. Our results indicate that the king oyster mushroom and its bioactive compounds (NAT and ERG) were able to decrease hallmarks of neuroinflammation and oxidative stress (OS) in microglial cells. Specifically, we showed that the king oyster mushroom, and particularly the extracts rich in NAT (K-AC and K-ET), along with pure NAT and ERG, had the strongest anti-inflammatory effects, which were similar to those of the positive control BB, showing promising effects on neuroinflammation. In the literature, studies with ERG have used concentrations ranging from 1 nM [32] to 10 mM [33] in endothelial or skin cells, with 500 μM [34,35] being the most used concentration. Our results using ERG to prevent nitrite production following LPS stimulation are in agreement with these data, in that ERG did not affect viability at the concentrations tested; therefore, we selected a concentration of 500 μM, which is in the range of blood ERG concentrations in healthy middle-aged and older adults (250 μM) [16]. On the other hand, K-ME-showed the highest cytotoxicity, with a decrease in viability starting at 0.2 mg/mL vs. 0.4 mg/mL for the other K extracts. A previous study [36], using ethanol (EtOH) and hot-water extracts of *K*, reported toxicity above 0.2 mg/mL. This is comparable to the range observed for our K-ME extract, but not for K-ET. However, differences in extraction procedures and cell viability assays between the two studies may account for these moderate discrepancies.

Nitrite (NO_2_^−^), which can be measured in cell culture media via the Griess assay, is the terminal product of NO oxidation. The latter is released from microglia following insult to the CNS or exposure to LPS [37]. Our data showed that the overproduction of nitrite induced by LPS-activated microglia was reduced by all king oyster extracts and the two bioactive compounds [37]. Furthermore, all of the extracts had a similar protective effect, as evidenced by the absence of statistical differences between the pretreatments and the positive control BB. The data with K-ET extract as a pretreatment are in accordance with the findings of a previous study showing an anti-inflammatory effect in LPS-treated BV2 microglia [36]. In this study, a protective effect was not observed with their aqueous extract [36]; this discrepancy with our crude extract (K) can be explained by their method of extraction with boiling water, which might have caused degradation of some of the fragile, active compounds that we might have conserved in our crude powder extract obtained at 50 °C.

The product of iNOS, NO°, is an important, highly diffusible biological messenger that plays a prominent role in the central nervous system, from the physiological to the pathological [21,37]. Its overproduction leads to oxidative stress, which causes significant damage to cell structures and biomolecular function, directly or indirectly leading to a number of neurological diseases [38]. Most of the studies reporting that mushrooms possess radical scavenging effects and a strong reducing power and ability to chelate ferrous ions are in vitro studies using extracts rich in polysaccharides [39]. Thus, the actions of the polysaccharides might explain the beneficial effects of our polar extract K-ME, although the concentration used here (0.1 mg/mL), which is closer to a dietary portion, is lower than the effective concentration of 10 mg/mL used in the previous study. This also highlights the efficiency of the bioactive contents of our extract with respect to the large difference in the doses of extract. Furthermore, the polysaccharides cannot explain the alleviating effects on nitrite production for the other K extracts, nor in the pure molecules ERG and NAT, because polysaccharides were not present. Therefore, decreases might be explained by a direct scavenger effect of pretreatments with the bioactive molecules ERG and NAT, and/or by a decrease in the production of NO° due to a decreased expression of iNOS.

Indeed, consistent with our results on nitrite, the iNOS expression was significantly downregulated when microglia were pretreated with K-AC, K-ET, and the crude powder K. The strongest effect was observed with K-AC; following this pretreatment, there was no difference between HAPI cells exposed or not to LPS stimulation, in a similar manner to the positive control BB.

The synergistic activation of iNOS expression with microglial NADPH oxidase (NOX) in inducing neuronal death is considered to be a key mechanism of inflammatory neurodegeneration [40,41]. In microglia, NADPH oxidase 2 (NOX2) has been identified as a major contributor to superoxide generation (O•) [41,42]. Therefore, targeting NOX2 overexpression could potentially improve the neuronal environment. As expected, LPS treatment strongly increased the expression of NOX2—a hallmark of a deleterious effect—but this overexpression was blunted in the microglia pretreated with K extracts (K-AC and K-ME) and NAT. The levels of NOX2 in pretreated microglia were no different than their controls and the positive BB extract.

Ergothioneine is claimed to be an antioxidant [12,34,35,43,44,45,46], which is consistent with our results, as assessed by a decrease in nitrite production. To the best of our knowledge, this is the first time that ERG has been evaluated to counteract microglia activation, which along with the other effects of ERG observed here, suggests that the effects of ERG are probably mediated by its actions on microglia, as described in [12]. We also observed an anti-inflammatory effect of ERG in preventing TNF-α release after LPS stimulation, which is consistent with the decrease in TNF-α gene expression reported in a recent study on mice treated with ERG [47] following LPS stimulation. This beneficial effect on TNF-α was similar to the effect with BB and with all treatments except for K-AC, which is surprising if we consider that the K-AC extract had a strong effect in reducing nitrite production. For the first time, we also showed a beneficial action of ERG, along with NAT and king oyster extracts (acetone, ETOH), as inhibitors of NRLP3 expression. Furthermore, we also described, for the first time, an effect of BB on the modulation of NLRP3 expression. As our results showed, the levels of NLRP3 expression were low in the control and pretreated microglia, while—as expected—LPS induced an overexpression of NLRP3 by microglia. According to the literature, the basal levels of NRLP3 expression are low, and the binding of LPS to TLR4 results in the activation of inducible NRLP3 and an increase in constitutive expression through the TLR4/NfkB signaling pathway [23,37,48] induced by ROS, also well known as one of the main triggers of NLPR3 inflammasome activation [24,43]. Here, we observed that the LPS-induced expression of NLRP3 was reversed by pretreatments with K-AC, K-ET, ERG, and NAT, as well as BB, supporting an anti-neuroinflammatory effect. In summary, taken as a whole, our results suggest that the reduction in ROS (assessed indirectly by nitrite production) observed with pretreatment with mushroom extracts or the pure NAT and ERG might be the first event to alleviate the activation of NLRP3. This decrease might be the result of a direct and/or indirect antioxidant effect via the concomitant decrease in NOX2 and iNOS expression mediated by the pretreatments. As is known, NLRP3 inflammasome activation is triggered by ROS through their binding to thioredoxin-interacting protein (TXNIP) [49]. TXNIP is able to sense the surge in ROS production and then dissociates from thioredoxin to bind NLRP3 and activate the NLRP3 inflammasome. This direct link establishes a sensory pathway between oxidative metabolism and inflammation. Therefore, pretreatment with mushroom extracts, ERG (with its high redox potential against ROS due to its original tautomeric state), or NAT (as an analog of the powerful antioxidant melatonin) might decrease the levels of ROS necessary to alleviate the interaction between TXNIP and NLRP3, thereby preventing NLRP3 overexpression. Moreover, oxidative stress is a well-established regulator of NF-κB and MAPK signaling and plays a critical role in the priming and activation of the NLRP3 inflammasome. During the priming step, NF-κB is activated downstream of the TNF-α, IL-1β, and Toll-like receptor (TLR) signaling pathways, leading to increased expression of NLRP3 [50]. The extracts and their bioactive compounds may influence upstream signaling pathways, including redox-sensitive transcription factors as well as key inflammatory pathways such as NF-κB and MAPKs, which are known to regulate microglial activation [50]. Although these mechanisms were not directly investigated here, our results provide indirect support for their involvement. Specifically, LPS-induced upregulation of TNF-α and NLRP3, together with their attenuation by K-AC and K-ET extracts and the bioactive compounds (ERG and NAT), as well as by the positive antioxidant control (blueberries), is consistent with the modulation of redox-sensitive signaling pathways mediated by antioxidant properties. However, further mechanistic studies will be required to directly assess the involvement of NF-κB, MAPKs, and related signaling pathways.

Interestingly, there is evidence indicating that the antidepressant-like actions of melatonin and serotonin, which are two structural analogs of NAT, are mediated through suppression of NLRP3 [51,52,53] expression after LPS activation in microglia [54]. Therefore, king oyster extracts rich in NAT (acetone and ETOH), as well as pure NAT, which decrease NLRP3 inflammasome expression, might mediate an antidepressant effect. Consistent with these observations, Park et al. [55] identified tryptamine, a structural analog of NAT, in *Pleurotus eryngii* (i.e., king oyster) and showed antidepressant activity similar to that of commercially available fluoxetine, also known as Prozac. Due to the structural similarities between tryptamine and NAT, with NAT having several advantages because of its physiological presence in blood [56] and its structure being very similar to that of serotonin and melatonin, we can expect that NAT might have a similar pharmacological advantage to tryptamine and be an interesting mushroom-based bioactive molecule, in addition to ERG. However, further studies in vivo are necessary to confirm whether mushroom extracts with the highest levels of NAT (K-AC) might exert antidepressant-like effects through the inhibition of microglial NLRP3 inflammasome activation. It is also worth noting that our positive control BB was also able to decrease NLRP3 expression, suggesting that BB, which has been shown to have positive effects on brain aging and on mood [57], might act through an NLPR3 mechanism. The effects of food ingredients in inhibiting NLRP3 expression and attenuating depressive-like behaviors induced by stress, including LPS, CORT, and reserpine, have previously been observed with a variety of natural antioxidant compounds, including quercetin [58,59]. Therefore, decreasing inflammation through NLRP3 modulation could participate as a molecular mechanism in mushrooms’ antidepressant effects [51] and their preservation of cognitive function in aging [25,60].

Our study has limitations, first, the HAPI microglial cell line provides valuable mechanistic insights and is widely used as an in vitro model of neuroinflammation because it retains key characteristics of primary microglia. However, although this model represents a reliable and practical model for investigating neuroinflammatory processes in vitro [37,61], it does not fully reproduce the complexity of the in vivo brain microenvironment, which could influence the observed effects of the mushroom extracts and their bioactive compounds. Second, the experimental conditions—such as exposure time, concentration of the extract, and the simplified cellular context—may not accurately reflect physiological or pathological concentrations and kinetics in the central nervous system. Third, while this study focused on microglial responses, neuroprotection in vivo involves complex interactions among multiple cell types, including neurons, astrocytes, and endothelial cells. The absence of these interactions limits the translational relevance of our findings. Moreover, the chemical composition of the mushroom extract, although characterized, may vary depending on source variability, which could affect reproducibility and biological activity. Finally, we cannot rule out a mushroom–microbiome interaction for generating secondary metabolites. Therefore, further studies using in vivo models are needed to confirm these preliminary results.

## 5. Conclusions

Our results indicate that the king oyster mushroom (*Pleurotus eryngii*), along with its components NAT and ERG, exerts anti-inflammatory effects in LPS-stimulated HAPI microglial cells, as evidenced by reduced nitrite and TNF-α production, as well as reduced expression of iNOS, NOX2, and NLRP3. Interestingly, the most efficient extract, K-AC, which was also the richest in NAT, produced similar results to the positive control (blueberry extract). The king oyster mushroom can therefore be highlighted as part of a healthy diet for its biological potential to fight neuroinflammation through NLRP3, and it might therefore improve mental health. These preliminary results are encouraging; however, further studies are needed to confirm the neuroprotective potential of the K-AC extract, NAT, and ERG, as well as to understand their mechanisms of action under more physiologically relevant conditions.

## Data Availability

The original contributions presented in this study are included in the article. Further inquiries can be directed to the corresponding author.

## Abbreviations

BB	Blueberry
CNS	Central nervous system
ERG	Ergothioneine
HAPI	Highly aggressively proliferating immortalized
INOS	Inducible nitric oxide synthase
K	King oyster
K-AC	King oyster extract in acetone
K-ET	King oyster extract in ethanol
K-ME	King oyster extract in methanol
NFκB	Nuclear factor kappa-light-chain-enhancer of activated B cells
NAT	*N*-acetyl tryptamine
NOX	NADPH oxidase
NOS	Nitric oxide synthase
Nrf2	NFE-E2-related factor 2
LPS	Lipopolysaccharide
TNF-α	Tumor necrosis factor-alpha
ROS	Reactive oxygen species
NLRP3	Nucleotide-binding oligomerization domain-like receptor family pyrin domain-containing-3
